# A Novel *In Vitro* Model to Study Pericytes in the Neurovascular Unit of the Developing Cortex

**DOI:** 10.1371/journal.pone.0081637

**Published:** 2013-11-21

**Authors:** Christoph M. Zehendner, Hannah E. Wedler, Heiko J. Luhmann

**Affiliations:** Institute of Physiology and Pathophysiology, University Medical Center of the Johannes Gutenberg-University, Mainz, Germany; Lerner Research Institute, United States of America

## Abstract

Cortical function is impaired in various disorders of the central nervous system including Alzheimer’s disease, autism and schizophrenia. Some of these disorders are speculated to be associated with insults in early brain development. Pericytes have been shown to regulate neurovascular integrity in development, health and disease. Hence, precisely controlled mechanisms must have evolved in evolution to operate pericyte proliferation, repair and cell fate within the neurovascular unit (NVU). It is well established that pericyte deficiency leads to NVU injury resulting in cognitive decline and neuroinflammation in cortical layers. However, little is known about the role of pericytes in pathophysiological processes of the developing cortex. Here we introduce an in vitro model that enables to precisely study pericytes in the immature cortex and show that moderate inflammation and hypoxia result in caspase-3 mediated pericyte loss. Using heterozygous EYFP-NG2 mouse mutants we performed live imaging of pericytes for several days in vitro. In addition we show that pericytes maintain their capacity to proliferate which may allow cell-based therapies like reprogramming of pericytes into induced neuronal cells in the presented approach.

## Introduction

Proper cortical development and function requires intact neurovascular coupling [1] and the intact brain endothelial barrier separating blood from the central nervous system (CNS) [1–3]. More than 70% of the cerebrovasculature in the rodent CNS is covered by pericytes [4]. This high percentage of microvascular pericyte coverage reflects their pivotal role for a proper formation of the blood-brain barrier (BBB), a highly organized vascular network within the CNS, which separates the neuronal parenchyma from peripheral circulation, and modulates its supply with nutrients. Besides pericytes various BBB transporters, tight junction proteins and cellular interactions including astrocytes [5], glia and neurons within the NVU orchestrate this complex task. Disturbances of this interplay significantly impair neurovascular integrity in the adult as well as developing brain and result in cognitive decline[2]. Bell et al. have recently demonstrated that pericyte loss impairs learning capability, results in neurovascular impairment and leads to accumulation of neurotoxic substances in the cortex of PDGFRb +/- mice starting at 1 month of age [4]. This finding points towards a crucial role of pericytes for proper cortical function. However, little is known about the role of pericytes in the immature cortex during pathological conditions. This is partly due to a lack of appropriate in vitro models that allow the analysis of pericyte-associated pathophysiology under precisely controlled in vitro conditions over prolonged time periods. It is speculated that infections and inflammatory processes during early brain development are implicated in a variety of neurological and psychiatric disorders including schizophrenia or autism [6–8]. The goal of the present study was to establish an in vitro model that allows to study pericytes in the developing cortex with a preserved neuronal network and to elucidate the impact of inflammation and hypoxia on pericytes.

Here we present a novel approach to study pericytes for days in cortical organotypic slice cultures (COSC) from newborn postnatal day 3-4 (P3-P4) mice with various methods including live cell imaging and electrophysiological recordings. Since the developmental stage of the CNS of newborn rodents at P3-P4 corresponds to that of preterm human babies at the age of postconceptional month 6 [9,10], our clinically relevant in vitro model allows to elucidate pericyte-associated cellular mechanisms in the developing cortex and to unravel processes of repair within the developing brain following e.g. inflammation or ischemia. It may also be a useful approach to assess cell-based therapies like reprogramming of pericytes into induced neuronal cells.

## Materials and Methods

### Ethics statement

All experiments were approved by the ethical committee of the “Landesungersuchungsamt Rheinland-Pfalz” and the authority “Landesuntersuchungsamt Rheinland-Pfalz”, protocol number: “Aktenzeichen 23 177 – 07 A12-1-004 and 23 177 – 07 A12-1-005”. Principles of laboratory animal care (European laws 86/609/EEC, national laws and NIH publication No. 86-23, revised 1985) were followed. All efforts were undertaken to minimize the number of animals used and their suffering.

### Preparation of COSC

Cortical organotypic slice cultures were prepared as described in detail before with slight modifications[11–13]. All efforts were made to minimize the number of animals used and their suffering. All experimental manipulations were carried out according to the European and national laws (86/609/EEC) on animal handling. In brief C57/BL6 or heterozygous EYFP-NG2 knockin P3/4 mouse pups were rapidly decapitated. The head was disinfected with a drop of ethanol 70% and the brain was quickly removed and transferred into 4°C cold medium. Beneath a benchtop microscope under laminar flow bulbi and the cerebellum were dissected. Hemispheres were carefully separated and meninges were removed with forceps. Afterwards hemispheres were cut into 350 µm thick coronal slices using a chopper. Cortices were transferred onto Millicell membrane filters (Merck Chemicals, Schwalbach, Germany) that were pre-equilibrated with medium over night at 37°C, 5 % CO_2_, humidified atmosphere. Medium was exchanged 1 day after preparation, thereafter every 2-3 days. Culture medium consisted of 50% MEM HEPES GlutaMax, 25% heat inactivated horse serum, 25% Hanks balanced salt solution supplemented with 1 mmol/l magnesiumchloride, 2 mmol/l calciumchloride. Glucose was added to a total concentration of 6-9 mg/ml, pH was adjusted to 7.2. We would like to stress the fact that pH was most important for the preparation and great care should be taken to make sure the pH is correctly adjusted to 7.2. For culture purposes Millicell filter membranes were pre-incubated in 6 Well plates containing 1 ml medium per well over night. COSC were carefully examined with light microscopy for intact morphology ahead of experimental manipulation. Only COSC that displayed an intact morphology were used for experimental procedures.

### 5-bromo-2'deoxyuridine (BrdU) application

BrdU was applied similarly as documented elsewhere[14]. In brief COSC were exposed towards medium containing 10 µmol/l BrdU for 3 hours on the third day in vitro. Subsequently COSC filter membranes were washed gently once and medium was switched to BrdU-free medium. COSC were fixed the following day in ice cold acetone (about 10 minutes) for immunohistochemistry. 

### Cardiac perfusion

C57/BL6 or heterozygous EYFP-NG2[15] mice were anesthetized by intraperitoneal (i.p.) injection of ketamine (120 mg/kg bodyweight) and xylazine (16 mg per KG bodyweight). Body temperature was kept at around 37°C with temperature controlled heating pads until the stadium of surgical tolerance according to Guedel[16] was reached. This was assessed by absence of a pain reflex upon toe pinch. The thorax was carefully opened with a microsurgical pair of scissors and animals were cardially perfused via the left ventricle with ice cold Ringer solution containing heparin (1 IU/ml). After removal of blood the perfusion solution was switched to 2% PFA and perfusion was continued for another 10 minutes. Brains were collected and post-fixed in 2 % PFA for 2 hours at 4°C. 100 µm thick coronal sections were cut with a vibratome and used for immunohistochemical processing. For some analyses brains were collected after blood was removed by saline perfusion and snap frozen in tissue tek with the help of liquid nitrogen. Here, 20 µm thick coronal sections were cut with a cryotome and fixed in ice cold acetone for 4-5 minutes. 

### Live Cell Imaging of pericytes

P3/4 heterozygous EYFP-NG2 mouse pups were deeply anesthetized by i.p. injection of ketamine (120 mg/kg bodyweight) and xylazine (16 mg per KG bodyweight). After surgical tolerance stadium was reached (tested as documented above) 50–100 µl tomato lectin were injected into the left ventricle and after 2 minutes animals were perfused with ice cold heparinized (1 IU/ml) Ringer solution. Thereafter brains were collected for COSC procedure as afore (see above) documented. COSC were examined with an upright confocal spinning disk system at 37°C in colorless Hanks balanced saline solution maintaining 1 mmol/l magnesiumchloride, 2 mmol/l calciumchloride and 6-9 mg/ml glucose.

### Immunohistochemistry

Immunohistochemical stainings were carried out according to standard procedures described in detail elsewhere[11,17]. Tables **1** and **2** give an overview on used antibodies, dilutions and purposes of the staining. In brief fixed probes were washed with 0.01 mol/l PBS. Subsequently tissue was blocked with 7% normal donkey serum (017-000-121, Dianova, Hamburg) and permeated with 0.3% triton in PBS 0.01 mol/l for two hours at room temperature. Only for BrdU staining samples were then incubated for 90 minutes at room temperature with 2 mol/l HCl. To verify the specific binding of the secondary anti-mouse antibody (used in Claudin-5 and NeuN stains) mouse IgG were blocked using a Fab (1:20, 2 hours in PBS 0.01 mol/l, AffiniPure Fab Fragment Donkey Anti-Mouse IgG (H+L), Jackson, Dianova) blocking technique. Primary antibodies were incubated in 2% bovine serum albumin (001-000-161 Diana, Hamburg) containing 0.05% azide and 0.1% triton in PBS 0.01 mol/l (overnight, room temperature). After incubation with primary antibodies probes were washed with PBS 0.01 mol/l and incubated with secondary antibodies and DAPI in 2% bovine serum albumin with 0.05% azide for another 2 hours at room temperature. After a final washing step in 0.01 mol/l PBS probes were embedded in Fluoromount. The sensitivity of the nitrotyrosine antibody was confirmed by positive controls with peroxynitrite as recommended by the manufacturer.

**Table 1:** Primary antibodies and stains.

**Antibodies and stains**	**Application**
anti-claudin 5 (Life technologies #35-2500), 1:50	endothelial marker, transmembraneous tight junction linker protein of the BBB and other endothelial cells e.g. within gut, kidneys
anti cleaved caspase-3 (Signaling Technology ASP 175, #9669), 1:200	Marker of apoptosis
anti-GFAP (Dakocytomation, Z 0334), 1:200	Marker of astrocytes
anti-NeuN (Millipore, MAB377), 1:200	Neuronal marker
anti-PDGFR beta (Neuromics, GT 15065), 1:200	Pericyte marker
anti-Ki67 (ab 15580 Abcam), 1:200	Cell proliferation marker
anti-BrdU, (347580, Becton Dickinson) 1:100	Staining against the nucleoside analogue of thymidine (BrdU), an indicator of DNA replication
Tomato lectin, TexasRed (Vector Laboratories)	Endothelial marker
DAPI (Sigma, 32670) 0.5 µg/ml	Cell nuclei staining
anti-Desmin (4024 Cell Signaling), 1:100	Pericyte marker
anti-pan-Laminin (ab7463, Abcam) 1:1000	Marker of basement membrane
Anti-Nitrotyrosine (Merck Millipore, 06-284), 1:200	Marker of oxidative stress induced by reaction of peroxynitrite with the amino acid tyrosine

**Table 2:** Secondary antibodies.

**Secondary antibodies**	**related primary antibodies**
Cy2 (A50-201C, Bethyl Lab, Biomol), 1:200	PDGFR beta (**Figure 1 C-F**, **Movie S1**)
Cy3 (705-165-147, Jackson, Dianova), 1:200	PDGFR beta (**Figures 1 G**, **2**; **3**; **4 C**; **5 B**; **6 A, E**, **Movies S3, S4, S5**)
DyLight 488 (711-485-152, Jackson, Dianova), 1:100 OR DyLight 488 produced in donkey (A120-208 D, Biomol), 1:100 – 1:200	BrdU (**Figure 5 A**, **Movies S5, S6**); Ki67 (**Figure 5 B**); cleaved caspase-3 (**Figure 7 A**), nitrotyrosine (**Figure 7 D, E**)
DyLight 488 (A90-337D2, Biomol), 1:200	claudin 5 (**Figures 2 A**, **6 B**, **Movies S3, S4**), NeuN (**Figure 2 B**)
Alexa Fluor 647 (711-605-152, Jackson, Dianova), 1:200	Desmin, NG2 (**Figures 1 C, F**, **4 E**, **Movies S1, S2**), GFAP (**Figure 2 A**, **Movie S2**), pan-Laminin (**Figures 3 A, B**, **Movie S4**)
Cy3 (715-165-151, Jackson, Dianova), 1:200	Claudin 5 (**Figures 1 B, E**; **3 E**)
DyLight 549 (712-505-153, Jackson, Dianova), 1:200	CD105 (**Figure 1 D**, **Movie S1**)

### Elecytrophysiological Recordings in Multi-Electrode Arrays

To evaluate spontaneous neuronal network activity of COSC after five days in vitro, MEA recordings were performed. COSC on Millicell filter membranes were cut out with a small scalpel and transferred on an 8 x 8 MEA chip (multichannel systems, Reutlingen, Germany) consisting of 60 electrodes with a diameter of 30 µm and an interelectrode distance of 200 µm. To avoid dislocation of COSC, a small weight was put on top of the slices. Prior to use the chip was heated up to a temperature of 37°C. COSC on MEA chips were kept at 20% O_2_ and 5% CO_2_ in a humidified atmosphere. Recordings were initiated 10 minutes after placing COSC on MEA chips and lasted 30 minutes. MC-Rack software version 4.1.1 (multichannel system, Reutlingen, Germany) was used for acquisition of the data. Sigma Plot version 7.0 (Systrat Software, Erkrath, Germany) was utilized for analysis. 

### Image analysis

All image analysis were processed and performed using Leica Application Suite Advanced Fluorescence (Leica Microsystems), Metamorph (Molecular Devices Corp., Downington, CA, USA), NIH Fiji is just ImageJ and IMARIS imaging software (Bitplane, Switzerland). Caspase-3 positive pericytes were identified by visual identification of PDGFR beta positive pericytes and cleaved caspase-3 co-localization. The ratio of pericytes positive for caspase-3 related to all pericytes was calculated within randomly chosen fields of view (RFV) of confocal images in COSC in layers II-IV. Images were obtained with a Leica SP5 confocal. The ratio of groups treated with IL-1 beta, hypoxia or both stimuli was set in relation to the mean ratio of respective control groups. Relative changes in fold change are presented. For this purpose 7-41 COSC preparations from 6-28 animals (up to 2812 pericytes) were analyzed for each condition. Microvascular pericyte coverage was determined by measuring total vessel length with the Fiji measurement tool in pixels similar as described before[11]. Total pericyte length was related to vessel length and relative microvascular pericyte coverage was obtained. For evaluation of caspase-3 inhibition on pericytes loss, pericytes in RFV of cortical layers II-IV were identified by DAPI, pan-laminin and PDGFR beta co-staining and the total number of pericytes per volume was determined via confocal z-stacks. Here the minimum spatial resolution was: 1.5 µm X * 1.5 µm Y * 2 µm Z voxel size. Values were related to control groups and differences in fold change are displayed. At least 3 COSC per group were analyzed.

If not stated otherwise all images shown in this manuscript are confocal images. 3 dimensional reconstructions of z-stacks were performed with IMARIS imaging software. 

### Image acquisition

We used a Leica SP5 confocal microscope for image analysis. Excitations were 405 nm, 488 nm, 561 and 633 nm. In addition we used an upright microscope with confocal spinning disk system (QLC10 Visitech, Sunderland, UK) equipped with a temperature controlled chamber for live cell imaging (excitation: 488 nm, 568 nm).

### Induction of hypoxia and inflammation, DPI and Z-DEVD-fmk treatment

Hypoxia was induced by placing COSC membranes into medium which was bubbled with a gas mixture of 8% O_2_, 5 % CO_2_, rest N_2_. Then probes were kept in a hypoxic incubator for 24 hours. Atmosphere in the incubator was adjusted to reach levels of about 75 mmHg O_2_ in culture medium. O_2_ levels were monitored ahead and after hypoxia using an Oxylite pO_2_ sensing probe (Oxford Optronics, Oxford, UK). For inflammation probes were incubated for 24 hours with medium containing 10 or 100 ng/ml Interleukin 1 beta (IL1B R & D Systems, Catalog number 401-ML). The inhibitor DPI was preincubated for 1 hour at a concentration of 50 µmol/l ahead of experimental manipulation. We chose this concentration because we found DPI to be protective at 50 µmol/l on brain endothelial cells during moderate hypoxic conditions (Zehendner et al. unpublished data). In addition DPI was successfully used at 50 µmol/l by other groups[18]. To elucidate impact of caspase-3 inhibition on pericyte cell death cells were treated with Z-DEVD-fmk (20 µmol/l) during 24 h of moderate hypoxic and inflammatory (100 ng/ml IL1B) conditions. Respective controls were treated with solvent (PBS, DMSO <0.1%) only. Hypoxic and / or inflammatory treatment was started on DIV 2 and COSC were fixed for analyses on DIV 3.

### Statistics

Results are documented as Mean + standard error of the mean (SEM). All data were analyzed using Graphpad Prism for Windows (version 4.02, Graphpad, San Diego, CA, USA). Datasets were checked for normalization by using D’Agostino and Pearson omnibus test. Groups that passed the D’Agostino and Pearson omnibus test[19] were therefore analyzed by two-sided unpaired student’s t-test. Groups that did not pass the test for normalization were analyzed with a two sided Mann-Whitney U test. Differences were regarded to be significant at alpha < 0.05.

## Results

### Perivascular cells within cortical organotypic slice cultures

**Figure 1:**
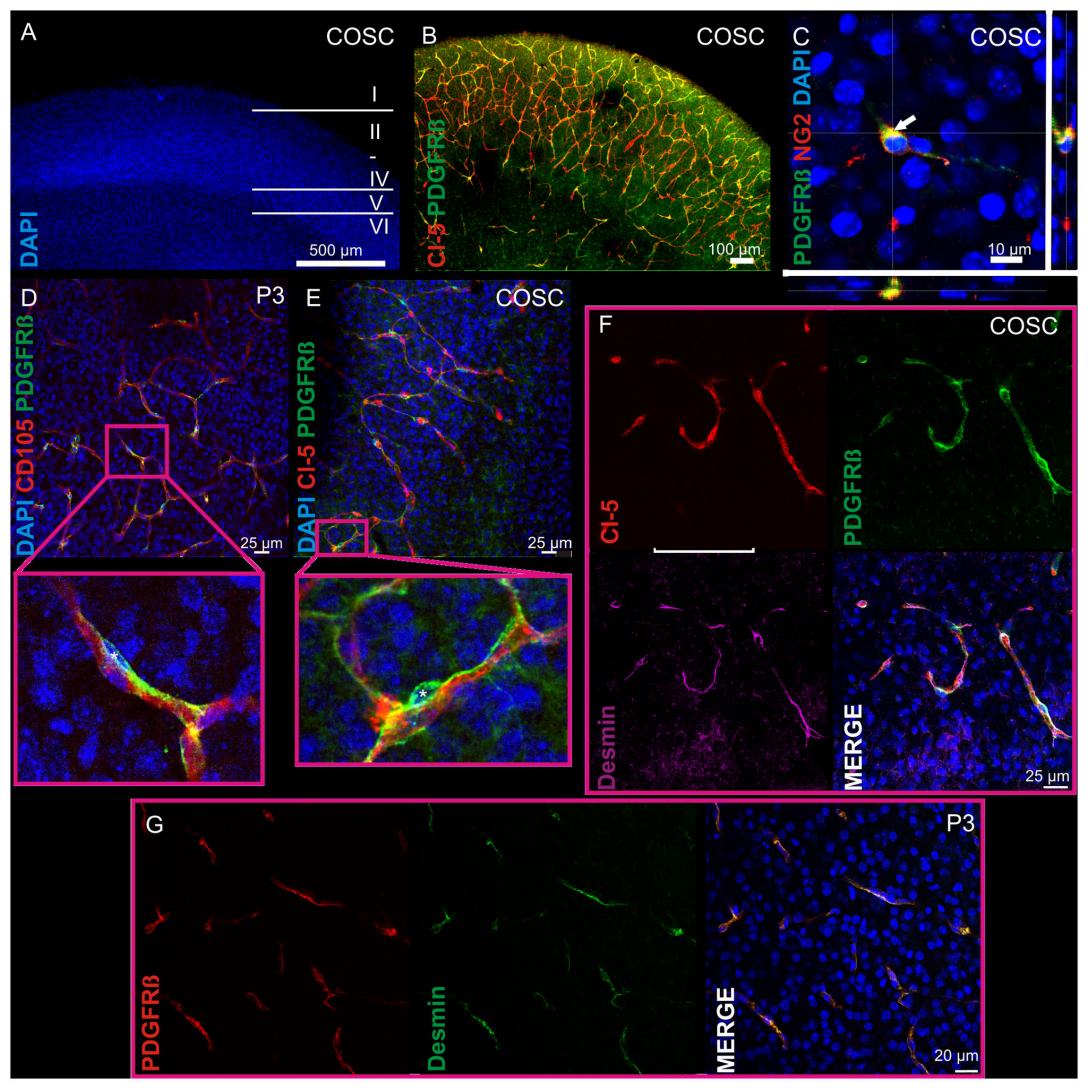
Pericytes in cortical organotypic slice cultures. Microvessels and pericytes in COSC were immunolabelled with different vascular markers (CD105, Cl-5), the pericyte marker PDGFR beta and DAPI. COSC preparations displayed a well preserved cytoarchitecture of cortical layers after 5 DIV (DAPI, epifluorescent image, A). A high number of microvessels covered by pericytes remained in COSC (epifluorescent image, B). PDGFR beta (green) partly co-localized (yellow) with the proteoglykan NG2 (red) in COSC which is another pericyte marker (arrowhead, confocal Z-stack with orthogonal section views, C). Similar to the perivascular localization of PDGFR beta positive cells in newborn mice at the age of postnatal day 3 (D) we observed PDGFR beta positive cells to be in close contact with microvessels in COSC after 5 DIV (**E**). Note the DAPI positive pericyte cell soma in which the nucleus is mostly spared by PDGFR beta staining (marked by asterisks) and its processes that follow the microvessel (D, E insets). Further the pericyte marker Desmin was found to be expressed by perivascular PDGFR beta positive cells in COSC (Panel **F**) as well as in native P3 mice (Panel **G**). Pericyte coverage in cortical microvessels was not significantly different in native P3 mice compared with COSC (**H**).

In the CNS the PDGFR beta receptor is exclusively expressed by pericytes and therefore represents a sensitive pericyte marker [4,20]. To identify perivascular PDGFR beta positive cells in COSC we performed immunohistochemical analyses with different vessel markers (CD105, Claudin 5), PDGFR beta and DAPI. Our COSC preparations displayed a well preserved cytoarchitecture of cortical layers after 5 days in vitro (DIV) as demonstrated by DAPI staining (Figure **1 A**). The transmembraneous tight junctional protein Claudin 5 (Cl-5) is expressed by brain endothelial cells [17] in COSC and is crucial for blood brain barrier integrity in vivo [21] and in vitro [12,17]. Cl-5 staining revealed a high number of Cl-5 positive microvessels within COSC that were in close contact with PDGFR beta positive cells (Figure **1 B**). Another protein expressed by pericytes is NG2. However NG2 is not solely restricted to pericytes but also expressed e.g. by oligodendrocyte precursor cells [22]. Co-stainings of PDGFR beta and NG2 demonstrated a partial co-localization of both pericyte markers (**arrowhead** Figure **1 C**) that was confirmed by 3 D reconstructions (Movies **S1, S2**). Pericytes in native P3 mice (Figure **1 D**) demonstrated a similar perivascular localization as found in COSC (Figure **1 E** **, insets**), demonstrating that our in vitro model recapitulates the anatomical in vivo situation. PDGFR beta positive cells appeared as cells with a DAPI positive cell soma, their processes following microvessels (Figure **1 D, E** **insets**). Note that the cell nucleus of a pericyte is mostly spared by PDGFR beta staining because PDGFR beta is a tyrosine kinase situated in the membrane of a pericyte, not in the nucleus [22]. The pericyte nucleus is surrounded by PDGFR beta staining that continues along pericyte processes. 

Another antigen reported to be expressed by pericytes is Desmin [22]. Desmin positive cells were found to co-localize with PDGFR beta next to cortical microvessels in COSC (Figure **1 F**). Desmin also co-localized with PDGFR beta in native P3 mice (Figure **1 G**). In addition microvascular pericyte coverage, a hallmark for neurovascular integrity [4], was not significantly different at 5 DIV within COSC compared with native P3 mice (COSC DIV5: 0.9 + 0.02 vs. P3: 0.89 + 0.02; n = 93 - 101 microvessels [>10 COSC preparations, 6 native P3 mice], P = 0.7821, Figure **1 H**).

### The neurovasular unit in the novel in vitro model

**Figure 2:**
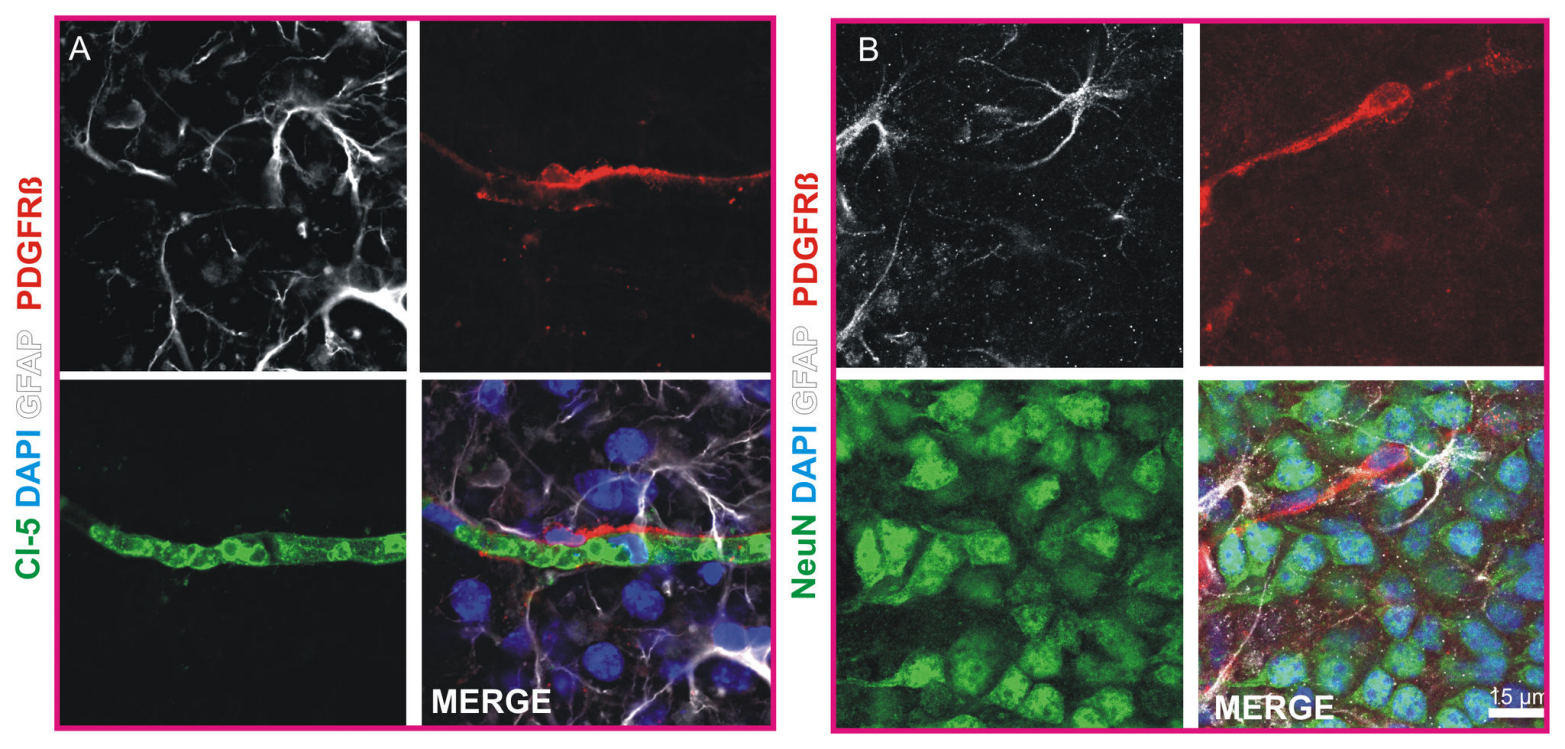
The neurovascular unit in COSC. Immunohistochemical stainings show a preserved morphology of astrocytes and pericytes that cover cortical microvessels in COSC (**A**). Stainings with the neuronal marker NeuN demonstrated a preserved neuronal morphology within the neurovascular unit of COSC (**B**).

**Figure 3:**
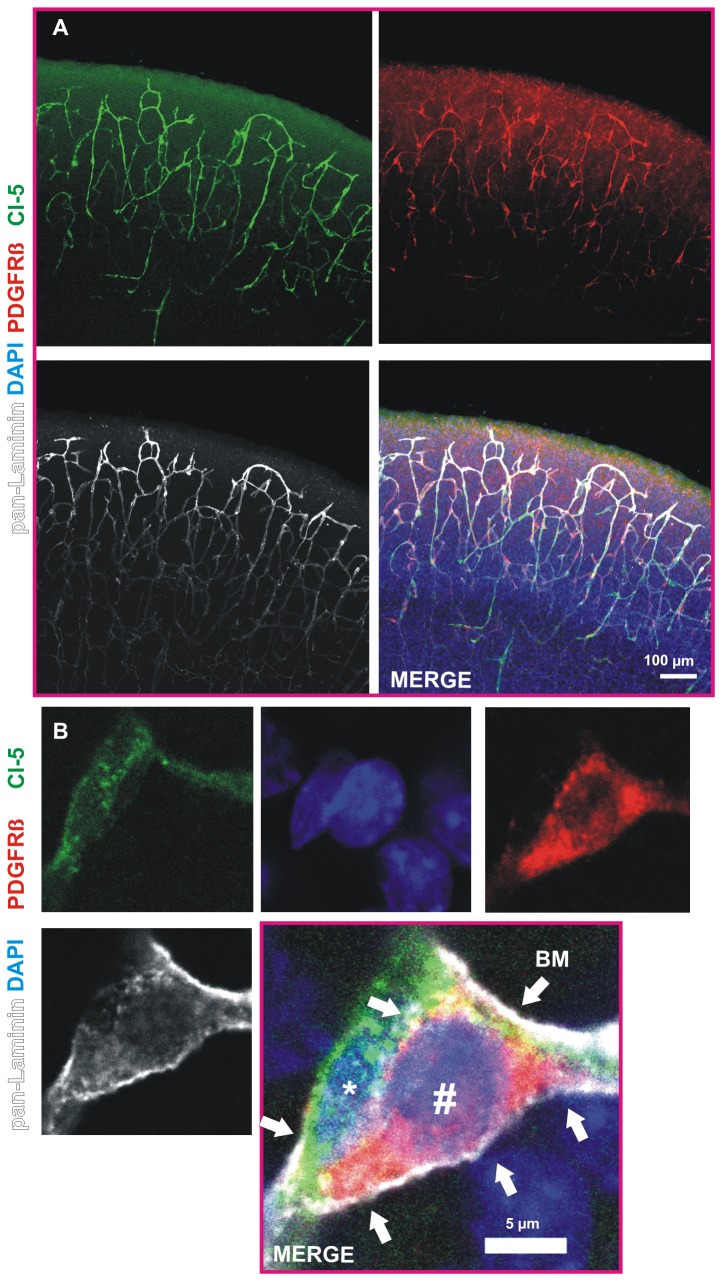
Basement membrane in the neurovascular unit of COSC. Co-labeling of cortical microvessels (Cl-5, green), pericytes (PDGFR beta, red) and laminins with a pan-Laminin antibody (white) reveals the presence of a basement membrane (BM) in the neurovascular unit in COSC after 4 DIV (confocal Z-stacks, maximum projection A). High power confocal magnification visualizes the BM (arrowheads, B) that encloses microvessels (Cl-5, green) and pericytes (PDGFR beta, red). Note the DAPI positive cell nuclei of the Cl-5 positive microvessel (asterisk) and the PDGFR beta positive pericyte (marked by #).

Immunohistochemical quadruple stainings show that in the presented in vitro approach almost all of the cell types of the neurovascular unit are present and display a preserved morphology in COSC after 3 DIV. Using high power imaging of cortical microvessels, pericytes and astrocytes we found that the endothelium is enwrapped by pericytes that are further in close contact with astrocytes (Figure **2 A**, Movie **S3**). NeuN immunoreactivity revealed the presence of neurons with an intact cellular morphology within the COSC preparation (Figure **2 B**). It has been reported that the basement membrane (BM), which consists of laminins, proteoglykans, nidogens as well as collagen IV [23], is of high relevance for neurovascular integrity [24,25]. Therefore we were interested in the question if a BM is present in the cortical neurovascular in vitro model. We found that cortical microvessels and pericytes in COSC possess a BM as demonstrated by pan-Laminin stainings (antibody that binds to Laminin chains alpha-1, beta-1, alpha-2 and gamma-1, and thereby many laminin isoforms that contain at least one of these chain types) in COSC [24,26]. Laminins are heterotrimers composed of an alpha, beta and gamma chain [23]. They are a hallmark for a proper formation of a BM and therefore are a marker of the BM in microvessels in the CNS [25,27]. We observed a preservation of the BM in the neurovascular unit of our in vitro preparation after 4 DIV (representative images from more than 10 COSC preparations, Figure **3 A**). High power confocal images revealed that cortical microvessels and pericytes are embedded in the BM (Figure **3 B**, Movie **S4**). Animated three-dimensional reconstructions with laser scanning confocal imaging confirmed these findings on an intact NVU (Movies **S3, S4**).

### Live cell imaging of pericytes in EYFP-NG2 knockin mice

**Figure 4:**
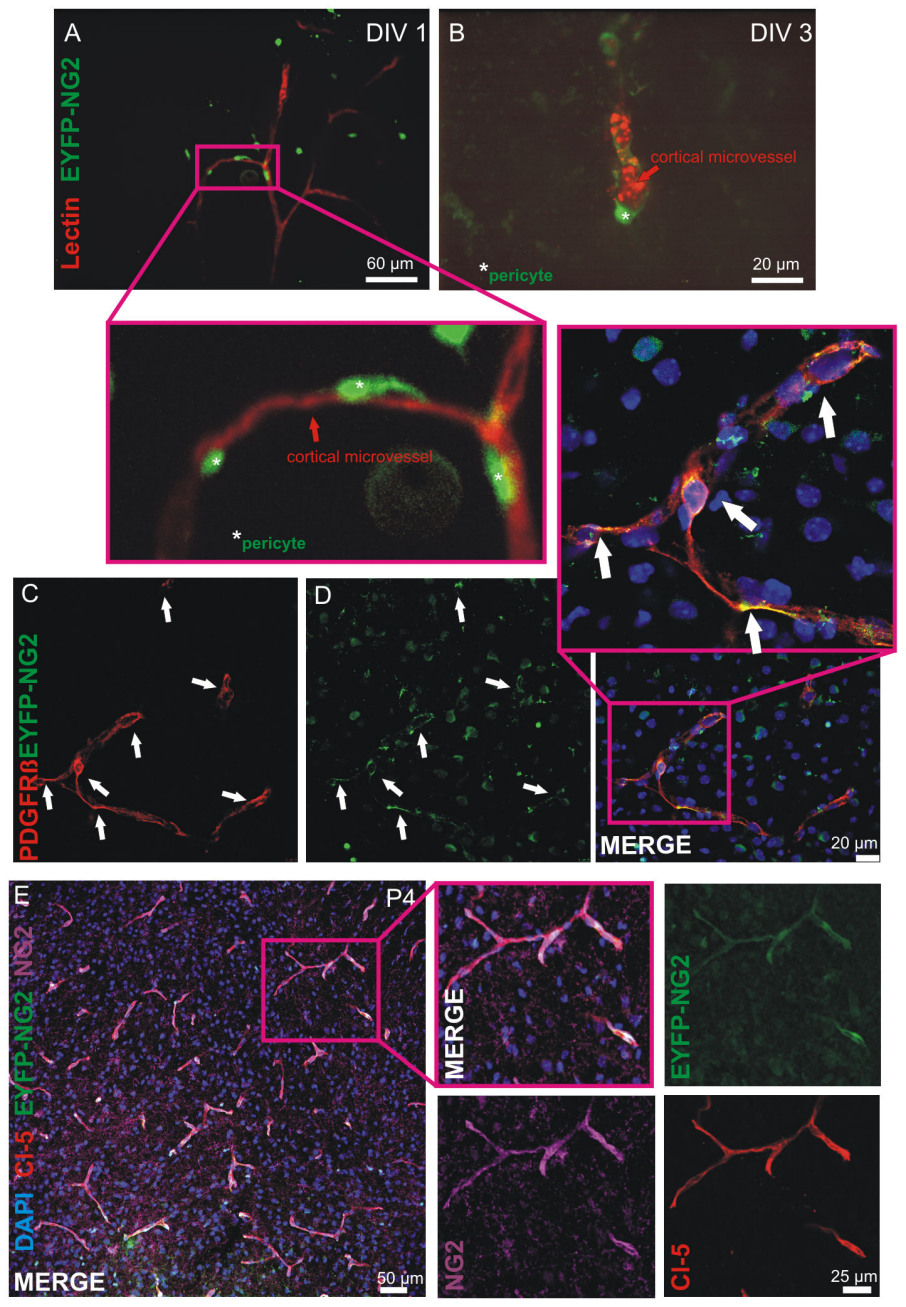
Live cell imaging of pericytes in COSC from EYFP-NG2 mice. Lectin stained microvessels in COSC appeared as red labeled vascular structures that were surrounded by EYFP-NG2 expressing pericytes (A, insets). Pericytes could be identified for up to 3 DIV 3 (**B**). Co-stainings with PDGFR beta (**C**, **D**), Cl-5 and NG2 demonstrate that perivascular EYFP-NG2 expressing cells are indeed pericytes (E, insets).

To address the question whether the in vitro model can be used for live cell imaging of pericytes, we labeled cortical microvessels of P4 heterozygous EYFP-NG2 knockin mouse mutants with tomato lectin. This mouse lineage carries an EYFP label inserted in exon 1 of the NG2 gene [28]. Here, in line with our immunohistochemical NG2 analyses, the EYFP-NG2 signal was partly present next to cortical microvessels in living COSC (Figure **4 A**). However other NG2 cells were not associated with microvessels indicating that they are not pericytes. Pericytes could be identified through the perivascular EYFP-NG2 signal for up to 3 days after COSC preparation (Figure **4 B**). To verify that the obtained perivascular EYFP-NG2 signals were of pericyte origin we performed immunohistochemical co-stainings of EYFP-NG2 cortices with PDGFR beta, NG2 and Cl-5, which demonstrated a co-localization of EYFP-NG2 and PDGFR beta in a perivascular manner (Figure **4 C, D** **, insets**). Co-stainings with NG2 and Cl-5 confirmed this observation (Figure **4 E**). 

### Pericytes in COSC are capable of proliferation

**Figure 5:**
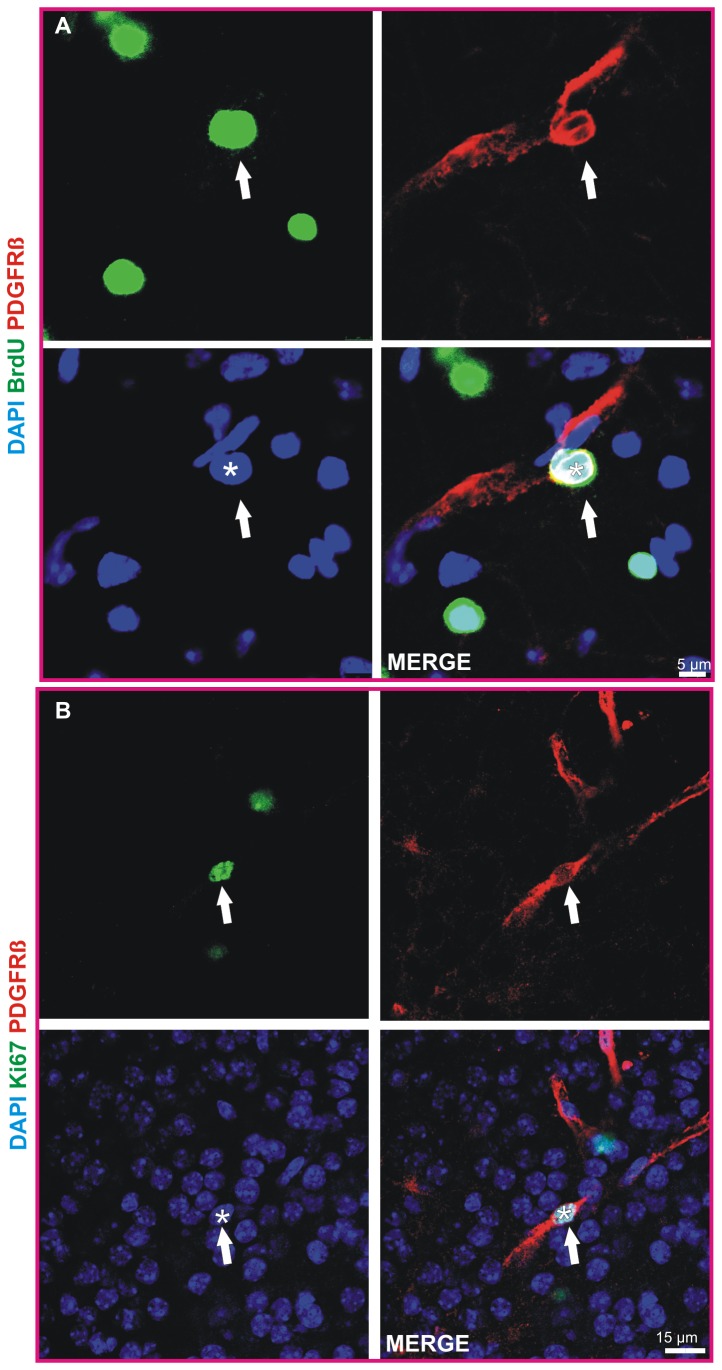
Pericytes in COSC are capable of cell division. Confocal analyses revealed that BrdU (3 hours exposition, 10µmol/l) was incorporated by pericytes within 24 hours on DIV 4 (arrowheads and asterisks in A mark a pericyte cell nucleus positive for BrdU). Ki-67 is a marker for cell proliferation. Here, a pericyte cell nucleus immunoreactive for Ki-67 on DIV 4 is shown (arrowheads, asterisks in B).

It has recently been shown that pericytes from human origin can be reprogrammed into induced neuronal cells by the use of vesicular stomatitis glycoprotein-pseudotyped retroviruses to transduce certain differentiation factors e.g. Mash1 and Sox2 [29]. However, this process is crucially dependent on the capacity of pericytes to proliferate because a successful transduction via retroviral vectors (except for lentiviruses) is dependent on the ability of the targeted cells for cell division. We found that BrdU (exposition for 3 hours on DIV 3, 10 µmol/l) was incorporated by some pericytes within 24 hours after exposure (Figure **5 A**, Movies **S5, S6**). In addition some pericytes were positive for Ki67 (Figure **5 B**), a marker for cell proliferation, after 4 DIV [30]. Ki67 and BrdU stainings were performed in more than 4 COSC preparations.

### Neuronal network activity and longterm survival of pericytes in vitro

**Figure 6:**
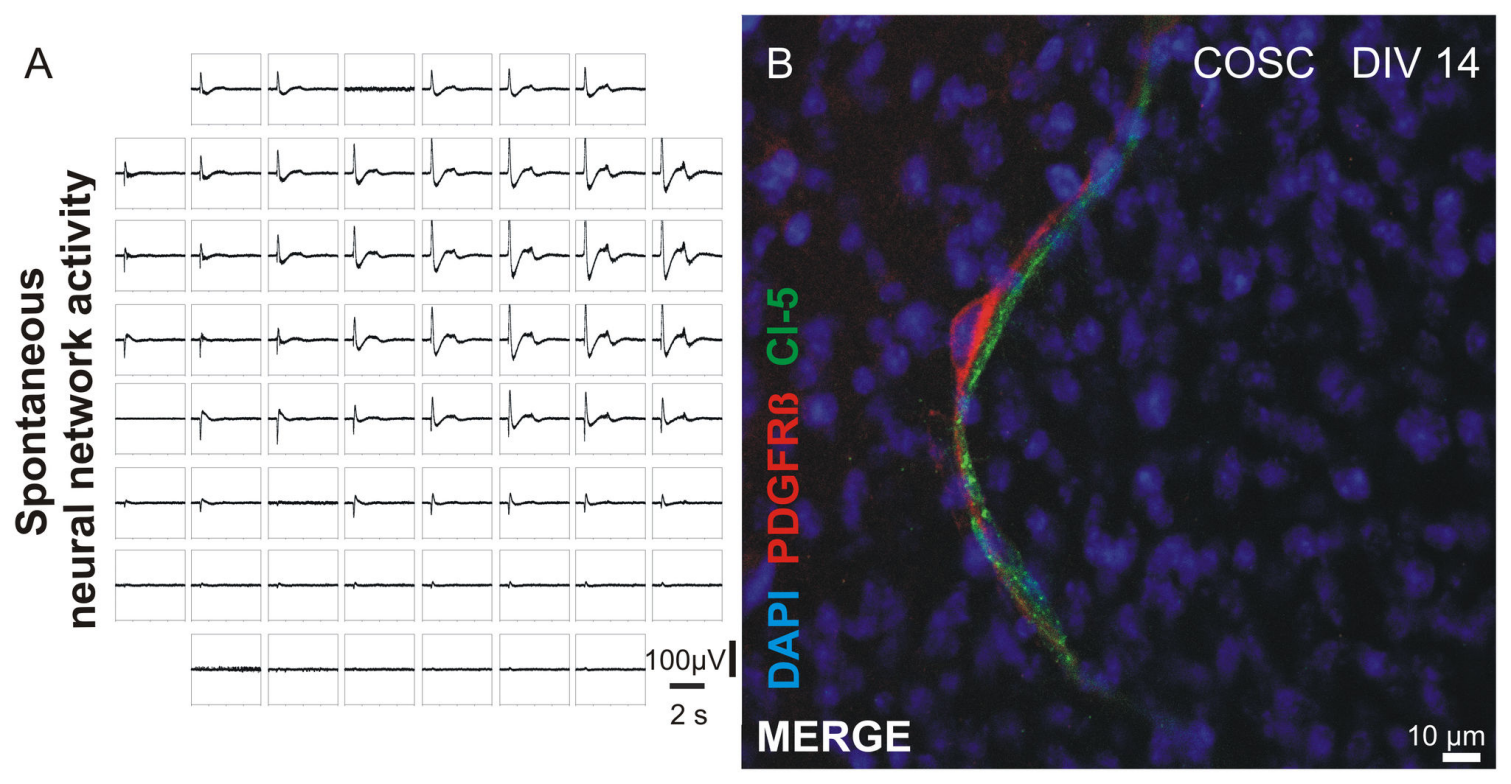
Spontaneous neural network activity and longterm persistence of pericytes in COSC. MEA recordings demonstrated that spontaneous synchronized neural network activity is preserved in COSC under culture conditions on DIV 5 (**A**). In addition pericytes were found to be present in COSC for weeks. Here, a 2 week old pericytes situated next to a Cl-5 positive microvessel is shown (**B**).

To elucidate if COSC maintain spontaneous network activity during culture conditions (5% CO_2_, 20% O_2_, rest N_2_, humidified atmosphere, 37°C) multi-electrode array (MEA) recordings were performed as described previously with slight modifications (Heck et al., 2008). Here we observed spontaneous synchronized neuronal network activity (Figure **6 A**, representative image from n = 3 independent recordings, DIV 5). Pericytes could be identified in COSC for up to 3 weeks. Figure **6 B** shows a pericyte with characteristic perivascular morphology after 14 days in vitro.

### Pericytes express elevated levels of caspase-3 after inflammation and hypoxia

Chronic hypoxia and inflammation have been proposed to be key factors leading to preterm brain injury [31]. In addition we and others have shown that caspase-3 is involved in neuronal and BBB pathology during ischemia and inflammation [17,32,33]. Therefore we were interested in the question if inflammation induced by interleukin 1 beta (IL1B), a cytokine which has been shown to disrupt proper white matter formation in the developing brain [34], and moderate hypoxia result in caspase-3 activation in pericytes. Thus we subjected COSC towards prolonged moderate hypoxia and different concentrations of IL1B. The cultures were exposed to a medium maintaining 71 + 2 mmHg pO_2_ for 24 hours. This pO_2_ level is about 58% of control medium kept in normoxic cell incubators (hypoxia 71 + 2.2 mmHg vs. normoxia 123 + 0.5 mmHg pO_2_, P < 0.0001, n = 15-48 measurements). 

**Figure 7:**
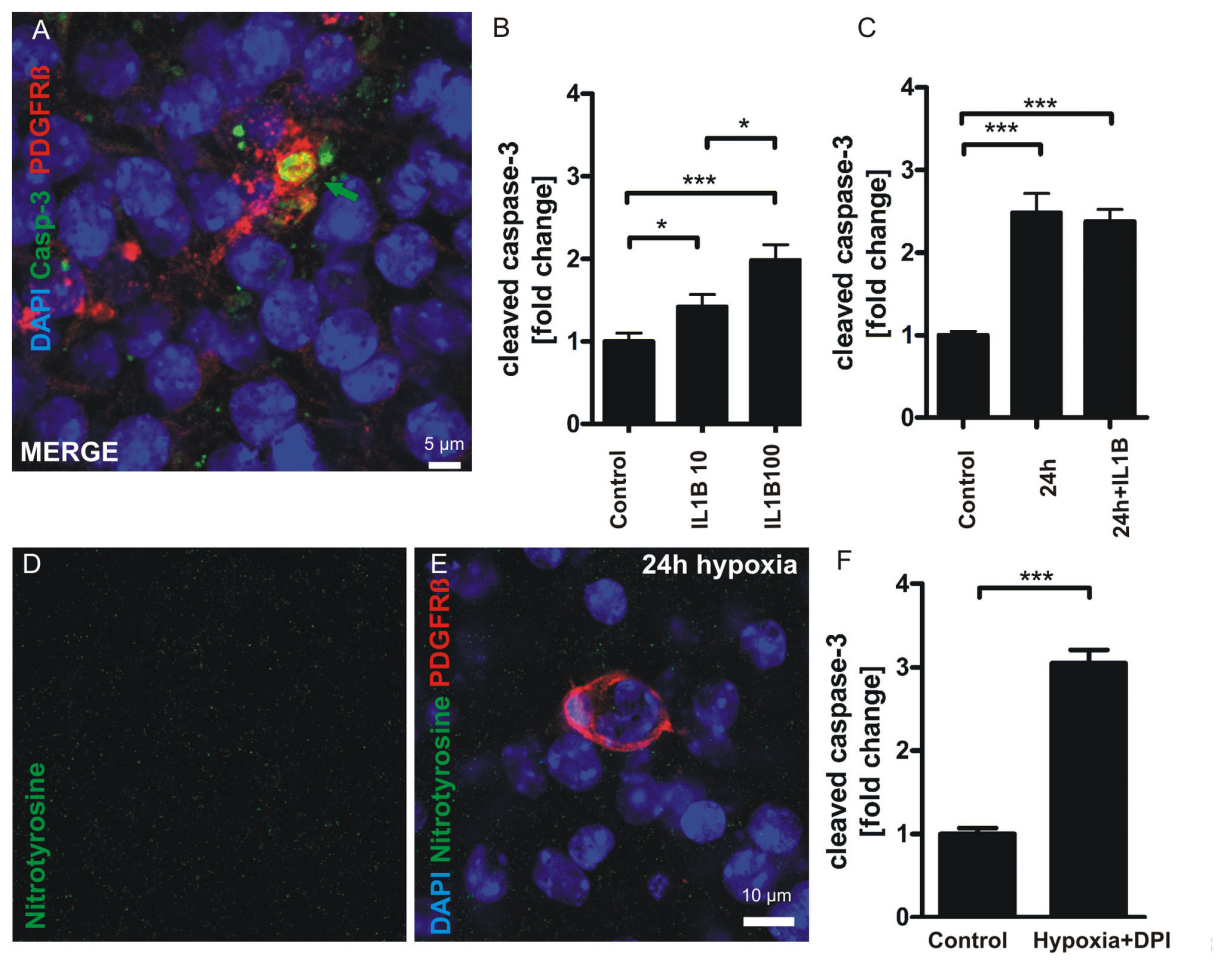
IL1B and moderate hypoxia induce caspase-3 cleavage in pericytes independently from peroxynitrite. Exposition of COSC towards pathologic conditions led to an increase of cleaved caspase-3 positive pericytes as shown in a representative confocal image in panel **A**. Stimulation of COSC with different concentrations of IL1B resulted in significantly elevated levels of cleaved caspase-3 in pericytes (**B**). Furthermore 24 hours of moderate hypoxia and a combination of IL1B and 24 hours of moderate hypoxia resulted in caspase-3 cleavage in pericytes (**C**). Of note, combination of IL1B and hypoxia did not significantly increase caspase-3 cleavage compared with probes treated with 24 hours of moderate hypoxia alone. Immunohistochemical stainings for nitrotyrosine did not reveal any significant formation of this ROS reaction product in pericytes (**D**, **E**). The NADPH oxidase inhibitor DPI [50µmol/l, 1 h pre-incubation] was not able to block caspase-3 cleavage under moderate hypoxic conditions. *P<0.05, ***P<0.001.

IL1B treatment resulted in significantly higher amounts of caspase-3 positive pericytes (representative image of a caspase-3 positive pericyte treated for 24 hours with IL1B 100 ng/ml, Figure **7 A**) after 24 hours compared with control (control: 1 + 0.1 vs. IL1B 10 ng/ml 1.42 + 0.15, P = 0.0269; control: 1 + 0.1 vs. IL1B 100 ng/ml 1.98 + 0.19, P < 0.0001, n = 7 - 8 COSC preparations per group [535-668 pericytes], Figure **7 B**). We detected significantly more caspase-3 positive pericytes in COSC treated with IL1B 100 ng/ml than in those treated with 10 ng/ml (IL1B 10 ng/ml 1.42 + 0.15 vs. IL1B 100 ng/ml 1.98 + 0.19, P = 0.0237, n = 8 COSC per group [535 - 668 pericytes], Figure **7 B**). 

Moderate hypoxia for 24 hours resulted in a significantly higher amount of cleaved caspase-3 positive pericytes (control: 1 + 0.05 vs. hypoxia: 2.48 + 0.23, P < 0.0001, n = 11-41 COSC preparations [571 - 2800 pericytes], Figure **7 C**). Exposure of combined hypoxia and IL1B 100 ng/ml significantly elevated cleaved caspase-3 levels in pericytes (control: 1 + 0.05 vs. yhpoxia+IL1B: 2.38 + 0.15, P < 0.0001, n = 18-41 COSC preparations [677-2800 pericytes], Figure **7 C**). However, a combination of IL1B and 24 h of moderate hypoxia did not increase caspase-3 cleavage compared with 24 h of moderate hypoxia alone (hypoxia: 2.48 + 0.23 vs. hypoxia+IL1B 2.38 + 0.15, P = 0.9763, n = 11 - 18 COSC preparations [571 - 677 pericytes], Figure **7 C**). 

It has been suggested that oxidative stress in form of reactive oxygen species (ROS) plays a major role in immature brain damage [31]. Therefore we were interested in the question if ROS may contribute to the observed enhancement of caspase-3 cleavage in pericytes after 24 h of hypoxia and whether an inhibition of oxidative stress may prevent caspase-3 cleavage. Stainings for nitrotyrosine, a product that arises from the reaction of the ROS peroxynitrite and the amino acid tyrosine [35] did not show any significant levels of nitrotyrosine in pericytes (representative image from at least 3 COSC preparations, Figure **7 D, E**). The NADPH oxidase is a major source of reactive oxygen species [36] in cerebral ischemia and diphenyliodonium chloride (DPI) is a widely used inhibitor of the NADPH oxidase [18]. We therefore tested if a pharmacological inhibition of NADPH oxidase by DPI has an effect on blockade of caspase-3 cleavage in pericytes during hypoxia. We found that DPI was not able to block caspase-3 cleavage in pericytes. Despite DPI treatment caspase-3 levels in the hypoxic group were significantly higher than in the normoxic control (hypoxia+DPI: 3.049 + 0.16 vs. normoxic control: 1 + 0.07, P < 0.0001, n = 24 COSC preparations per group [1597-2812 pericytes], Figure **7 F**). 

### Caspase-3 inhibition ameliorates pericyte loss in hypoxia and inflammation

Because we observed enhanced levels of cleaved caspase-3 in pericytes upon ischemia and inflammation we were interested in the question if caspase-3 activation may result in pericyte loss. To address this question COSC were treated with the selective and irreversible caspase-3 inhibitor Z-DEVD-fmk [17] during hypoxia and inflammation and pericyte numbers in confocal z-stacks were compared with solvent treated normoxic and non-inflammatory control COSC. We found that hypoxia resulted in a significant decrease of pericytes/volume compared with controls (control: 1 + 0.16 vs. hypoxia 0.31 + 0.02, P = 0.0034, n = 5 - 6 RFV from more than 3 COSC). After IL1B (100 ng/ml) treatment pericytes were also significantly reduced in cortical layers II-IV (control: 1 + 0.16 vs. IL1B 0.46 + 0.05, P = 0.0142, n = 5-6 RFV from more than 3 COSC preparations per group). COSC that were treated with Z-DEVD-fmk sustained significantly higher pericyte numbers after hypoxic (hypoxia 0.31 + 0.02 vs. hypoxia/Z-DEVD-fmk 0.52 + 0.08, P = 0.0324, n = 5 RFV from more than 3 COSC) and inflammatory conditions compared with solvent treated groups (IL1B 0.46 + 0.05 vs. IL1B/ZDEVD-fmk 0.95 + 0.16, P = 0.0406, n 5 - 8 RFV from more than 3 COSC preparations per group). However Z-DEVD-fmk was not able to completely block pericyte loss under hypoxic conditions (control: 1 + 0.16 vs. hypoxia/Z-DEVD-fmk 0.52 + 0.08, P = 0.0301, n = 5 - 6 RFV from more than 3 COSC).

## Discussion

In this report we demonstrate that cortical organotypic slice cultures from neonatal rodents are a useful model to study pericytes within the intact neurovascular unit under various experimental conditions. Further we show that pathological conditions such as moderate hypoxia and inflammation result in caspase-3 mediated pericyte loss.

To our knowledge no other experimental technique has yet been described that allows the investigation of pericytes in vitro for days including live cell imaging in a preserved neurovascular environment. In line with our data Kovacs and colleagues have shown that it is possible to study neurovascular interactions in slice cultures of the hippocampus of the neonatal rat [37] for days in vitro. Other in vitro approaches that imply pericytes are mostly used to assess BBB permeability during various conditions [38]. Such approaches do not allow any investigations on pathophysiological mechanisms in the immature cortex because an intact neural network is missing. However, the presented approach has certain limitations. Our in vitro setting lacks cerebral blood flow. Shear stress in vessels has been shown to be important for a proper formation of the BBB [39]. Therefore our model does not allow the analysis of shear stress effects on pericytes. This issue may be best addressed by in vivo investigations because the pulsatile character of cerebral blood flow including the composition of the different cellular blood components is difficult to reflect in vitro. Further live cell imaging with the presented lectin perfusion is not feasible for several weeks but rather limited to a couple of days. The reason herefore is that the lectin signal vanishes over time because the dye is injected into circulation on the day of preparation. 

Our data show that most of the cellular components of the NVU are morphologically preserved in vitro including a basement membrane which is an anatomical key feature for pericyte identification [40]. It has been suggested that e.g. during depletion of VEGF microvessels vanish, but the basal laminina remains [40]. Our laminin stainings indicate that microvessels in cortical layers II-IV did not significantly decrease within DIV 3-5 as nearly all BM tubes labeled by laminin were associated with Cl5 positive microvessels. We would like to stress that the presented model should be used within 3-5 DIV for quantitative pericyte analyses because we focused on this time period in our setting. We were not able to distinguish an endothelial from a parenchymal BM with pan-Laminin stainings, which is in line with data from Sixt et al. [25]. Endothelial and parenchymal BM only become distinguishable from each other in conditions such as inflammation but not in physiological states. Therefore our model may also allow detailed analyses on the involvement of the BM during pathological neurovascular conditions. We have used a set of pericyte, BM and vascular markers to be sure that PDGFR beta positive perivascular cells are indeed pericytes. Stainings for the pericytes markers NG2, PDGFR beta and Desmin partly co-localized and varied in staining patterns. This is in good agreement with previous reports [22]. The discrepancy e.g. found in Desmin and PDGFR beta staining is due to the fact that Desmin is a cytoskeleton protein which is situated intracellular while PDGFR beta is a tyrosine kinase located on the pericyte’s cell surface (for a detailed review see for example 22. In addition our electrophysiological recordings from COSC on MEA arrays demonstrate that spontaneous neuronal network activity is also preserved during incubator conditions and while recording in culture medium. We would like to stress this fact because usually MEA recordings in COSC are performed using artificial cerebrospinal fluid perfusion containing 95 % oxygen and 5 % CO_2_ [33]. 

Another goal of the present study was to establish a protocol for pericyte live cell imaging in the developing cortex in vitro using a transgenic mouse line. In line with Karram et al. [28] we have shown that the EYFP-NG2 signal co-localizes partly with the pericyte marker PDGFR beta in a perivascular manner and verified that the EYFP signal is restricted to NG2 by the use of NG2 antibody staining. By lectin perfusion of the brain microvasculature we demonstrate that pericytes can be monitored live in vitro. Live cell imaging of pericytes within the neurovascular unit may allow e.g. evaluation of the timing of pericyte constriction during hypoxia and/or inflammation. We would like to outline that for this purpose other genetically modified mouse lines e.g. NG2DsRedBAC created by Zhu and colleagues [22,41] may also be useful.

**Figure 8:**
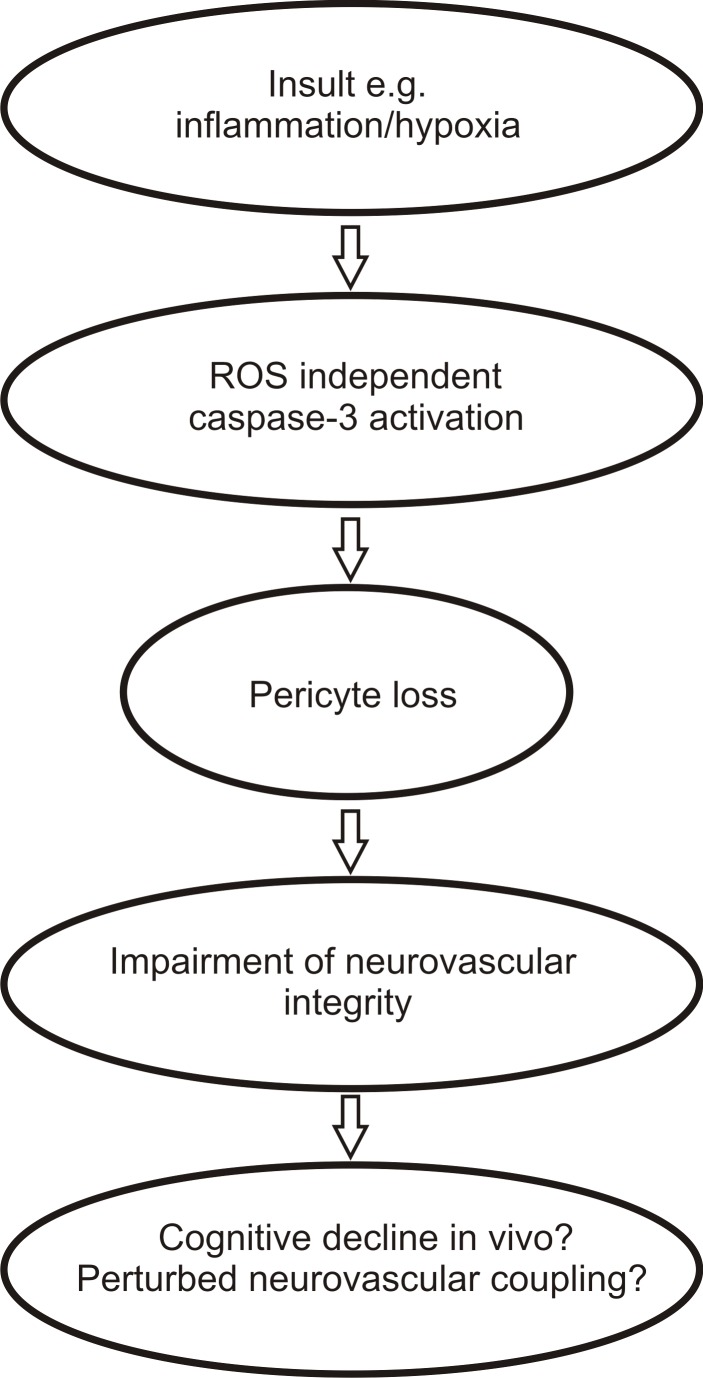
Pericyte impairment in the developing cortex during pathologic conditions. Hypoxia and inflammation result in caspase-3 dependent loss of pericytes in cortical layers. Pericyte loss may be of relevance for perturbances in neurovascular integrity in the developing brain and may be involved in a variety of pathologic sequelae e.g. cognitive decline in ageing or neuroinflammation.

Hypoxia and inflammatory stimuli are hallmarks in the pathogenesis of immature brain damage [10]. The resulting encephalopathy of the premature is a severe brain injury with a heterogeneous phenotype [31]. Epidemiologic studies point to a relation of infections in prematurely born infants with disorders like schizophrenia, autism [7,8,42] and later cognitive impairment [43]. These pathologies are associated with cortical gray matter changes [44,45]. In addition clinical studies have demonstrated a relation between premature brain injury and cerebral blood flow (CBF) regulation: perturbances of CBF autoregulation is a predictor of severe brain injury in preterm babies [46]. However, the exact cellular mechanisms underlying these phenomena are not fully understood. Peppiatt et al. [47] demonstrated that pericytes are capable of modulating the diameter of brain microvessels in slice preparations of the cerebellum. Further a loss of pericytes is associated with the formation of cerebral microaneurysms [48]. These findings and recent data of Bell et al. who have shown that pericyte deficiency leads to neurovascular impairment and consecutive neuroinflammation in cortical layers [4], point to a significant role of pericytes in regulating the NVU that is important for cortical function throughout embryogenesis and further development. Therefore another goal of the present study was to evaluate the consequences of hypoxic and inflammatory conditions on pericytes in the developing cortex. We demonstrate that pathological stimuli e.g. moderate hypoxia over 24 hours and sterile inflammation by IL1B, result in an increased level of cleaved caspase-3 activity within pericytes. We have previously shown that caspase-3 contributes to rapid anoxic neurovascular unit damage (RANUD) [17] and that in neonatal mice caspase-3 is essential for proper cortical development [49], as already demonstrated before [50]. Furthermore it has been recently documented that inhibition of caspase-3 reduces cortical lesions and improves neurological outcome in a model of neonatal hypoxia-ischemia brain damage in rodents [51]. These data point to a crucial and precisely regulated role of caspase-3 in physiology and pathophysiology of the developing cortex. In line with other groups [51] our experiments show that caspase-3 cleavage is present in hypoxic and inflammatory conditions in the immature cortex. While it has been shown that a combination of inflammation and hypoxia potentiates cortical damage [52], we did not find an increase of caspase-3 cleavage in pericytes upon combined inflammation and moderate hypoxia. However our experimental setting differed from previous studies that showed an increase of brain injury under these double hit conditions. For instance Brochu and colleagues [52] induced hypoxia by exposing neonates to 8% O_2_ after ligation of one common carotid artery and injection with lipopolysaccharide (LPS), a surface protein of gram-negative bacteria that occur for example during severe sepsis. These hypoxic and inflammatory stimuli are much more severe than moderate hypoxia and treatment with IL1B as used in the present study. Another explanation could be that the neurodegenerative mechanism of LPS may be mediated by direct activation of the brain endothelium and independently from IL1B as shown by Murray et al. [53]. However it has recently been proposed that moderate inflammation by IL1B and moderate hypoxic conditions are clinically more relevant for the pathogenesis of brain injury of prematurely born infants [54]. Our results indicate that caspase-3 cleavage in pericytes increases in a dose dependent manner. Even rather low concentrations of IL1B (10 ng/ml) were capable of inducing significant caspase-3 activation in pericytes which points to a critical vulnerability of pericytes in the developing cortical neurovascular unit. Our results indicate that hypoxia and inflammation lead to caspase-3 activation which results in pericyte loss. A pharmacological inhibition with a specific caspase-3 inhibitor support this hypothesis. Pericyte loss has been shown to result in BBB impairment and causes an accumulation of neurotoxic substances in the cortical parenchyma in vivo [4]. We suspect that the detected pericyte loss in our experimental conditions may have major implications for pathophysiological processes in vivo within the developing cortex (Figure 8) and may result in e.g. accumulation of neurotoxins that may cause cognitive decline in ageing [4]. We suggest that caspase-3 inhibition may be a promising target with regard to stabilization of the NVU by protecting pericytes in inflammatory and hypoxic states. This finding may also have implications for other parts of the CNS e.g. the spinal cord. Hypoxic brain damage and inflammation have been demonstrated to be predominantly relevant in white matter impairment in the developing brain [9,34]. However our data show that caspase-3 activation in the immature cortex under hypoxia and inflammation leads to pericyte loss. These findings point out that pericytes may be another cell population which may be significantly affected by ischemic and inflammatory insults in the developing cortex.

Recently it has been shown that pericytes prevent reperfusion of microvessels after MCAO in an oxidative stress dependent manner. Here, the reactive oxygen species peroxynitrite was shown to be of major importance [35]. In our model we did not find elevated levels of nitrotyrosine in pericytes after 24 hours of moderate hypoxia. Furthermore an inhibitor of the NADPH oxidase (DPI) was also unable to block the observed caspase-3 activation in pericytes. Therefore we conclude that peroxynitrite does not have a major role for caspase-3 activation in pericytes during 24 hours of hypoxia without reoxygenation. However, in our setting we evaluated the effect of moderate hypoxia without reperfusion. This is a significantly different condition than the setting of Yemisci and colleagues who have investigated the effect of middle cerebral artery occlusion followed by reperfusion [35]. However, prolonged episodes of hypoxia in the newborn brain are thought to better reflect the in vivo situation [9] because the majority of prematurely born infants do not suffer from focal ischemia, e.g. an occluded cerebral vessel, but rather from insufficient blood oxygenation due to immature lungs and an immature breathing center within the brain stem [55]. 

Our results on the capacity of pericytes to proliferate in vitro may represent a promising therapeutical strategy. It has been shown that pericytes are involved in central nervous scar formation [56]. In addition pericytes may be a target to compensate for neuronal injury [29]. Karow and colleagues have shown that human pericytes can be reprogrammed into induced neuronal cells [29]. These authors used a retroviral vector to transduce differentiation factors which requires that targeted cells are capable of cell division [29]. BrdU and Ki-67 stainings show that some pericytes in our experimental approach remain their capacity to proliferate. Hence we speculate that the presented technique may lead to protocols for the reprogramming of pericytes into neuronal cells in a preserved neurovascular unit within the cortex. We hypothesize that such protocols may also be of use for regenerative therapies in order to compensate for neuronal injury in the cortex e.g. stroke or dementia. In addition we found that pericytes remain in COSC for up to three weeks which gives the opportunity to perform e.g. long term fate mapping of pericytes. However this will of course require different transgenic mouse models than we used in our experiments (see for example 56.

## Supporting Information

Movie S1 **Here, an animated confocal 3 D Z-stack (thickness of planes 700nm) of a quadruple staining of pericytes, cell nuclei and microvessels is shown.** White: NG2 a marker for pericytes, Red: vascular marker CD105, Green: PDGFR beta, a pericyte marker, DAPI in blue: stains cellular nuclei. Note that NG2 and PDGFR beta partly co-localize in a perivascular manner indicating that PDGFR beta positive perivascular cells are indeed pericytes.
(AVI)

Movie S2 **To better appreciate the NG2 (white) signal within the perivascular pericyte channels representing the microvessel (red) and PDGFR beta signal (green) from Movie S1 have been removed in this reconstruction.** Note the cell nucleus of the NG2 positive pericyte in the center of the animation that is in close contact with the NG2 signal.
(AVI)

Movie S3 **A 3 dimensional reconstruction (confocal Z-stack, thickness of planes: 700nm) of the neurovascular unit.** Red: PDGFR beta positive pericyte which covers a cortical microvessel (labeled with Cl-5, green). Note the close relationship of astrocytotic endfeet (GFAP, white) that surround the pericyte. An animated fly-through the vessel demonstrates that PDGFR beta is partly co-localizing (yellow) with Cl-5 which is due to the fact the PDGFR beta is a tyrosine kinase located at the cell membrane while Cl-5 is a transmembraneous tight junction linker protein.
(AVI)

Movie S4 **An animated 3 D reconstruction (confocal Z-stack, thickness of planes: 700nm) of the neurovascular unit.** Red: PDGFR beta positive pericyte next to a cortical microvessel (green, Cl-5). Note that both vessel and pericyte are enclosed by laminin (pan-laminin labeling, white) which indicates the presence of a basement membrane.
(AVI)

Movie S5 **Here a cell nucleus (DAPI, blue) positive for BrdU (green) of a pericyte (labeled with PDGFR beta in red) is shown in an animated confocal 3 D Z-stack (700nm thickness of planes).** Note that the cell membrane of the pericyte stains positive for PDGFR beta (red) whilst its cell nucleus is spared due to the localization of PDGFR beta on the cell membrane of the pericyte.
(AVI)

Movie S6 **To better demonstrate the pericyte’s BrdU positive cell nucleus from Movie S5 all channels except the green channel (BrdU) have been removed in this 3 D reconstruction.**
(AVI)
